# Adenosquamous carcinoma arising within a long‐standing intrapulmonary bronchogenic cyst in an adult presenting with hyponatraemia

**DOI:** 10.1111/cyt.13446

**Published:** 2024-09-23

**Authors:** Adeyinka Akinsanya, Sheila Segura, Harvey Cramer, Hector Mesa

**Affiliations:** ^1^ Department of Pathology and Laboratory Medicine Indiana University School of Medicine Indianapolis Indiana USA

**Keywords:** adenosquamous carcinoma, bronchogenic cyst, hyponatremia, neoplastic cell transformation, non‐small cell lung carcinomas

## Abstract

A 74‐year‐old woman's persistent hyponatraemia led to the discovery of an adenosquamous carcinoma within an intrapulmonary bronchogenic cyst (IPBC), diagnosed 59 years prior. This is the first reported case of such a transformation in an IPBC.

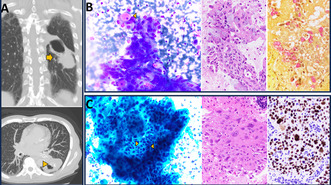

An adenosquamous carcinoma, originating from an intrapulmonary bronchogenic cyst identified 59 years prior, was discovered during the workup for a patient's unexplained, persistent hyponatraemia.

## 1 INTRODUCTION

Bronchogenic cysts (BC) are rare congenital bronchopulmonary foregut malformations that occur due to defective budding during the early development of the tracheobronchial tree.^1^ Most are in the mediastinum, and a third are intrapulmonary (IPBC). Clinical presentation ranges from asymptomatic to symptoms due to compression of adjacent structures, infection, rupture and very rarely malignant transformation. Reported subtypes of malignant transformation to date include adenocarcinoma, squamous cell carcinoma, anaplastic carcinoma, and mucoepidermoid carcinoma.^2^, ^3^ We herein report the first case of adenosquamous carcinoma (ASC) arising from an IPBC.

## 2 CASE HISTORY

**FIGURE 1:**
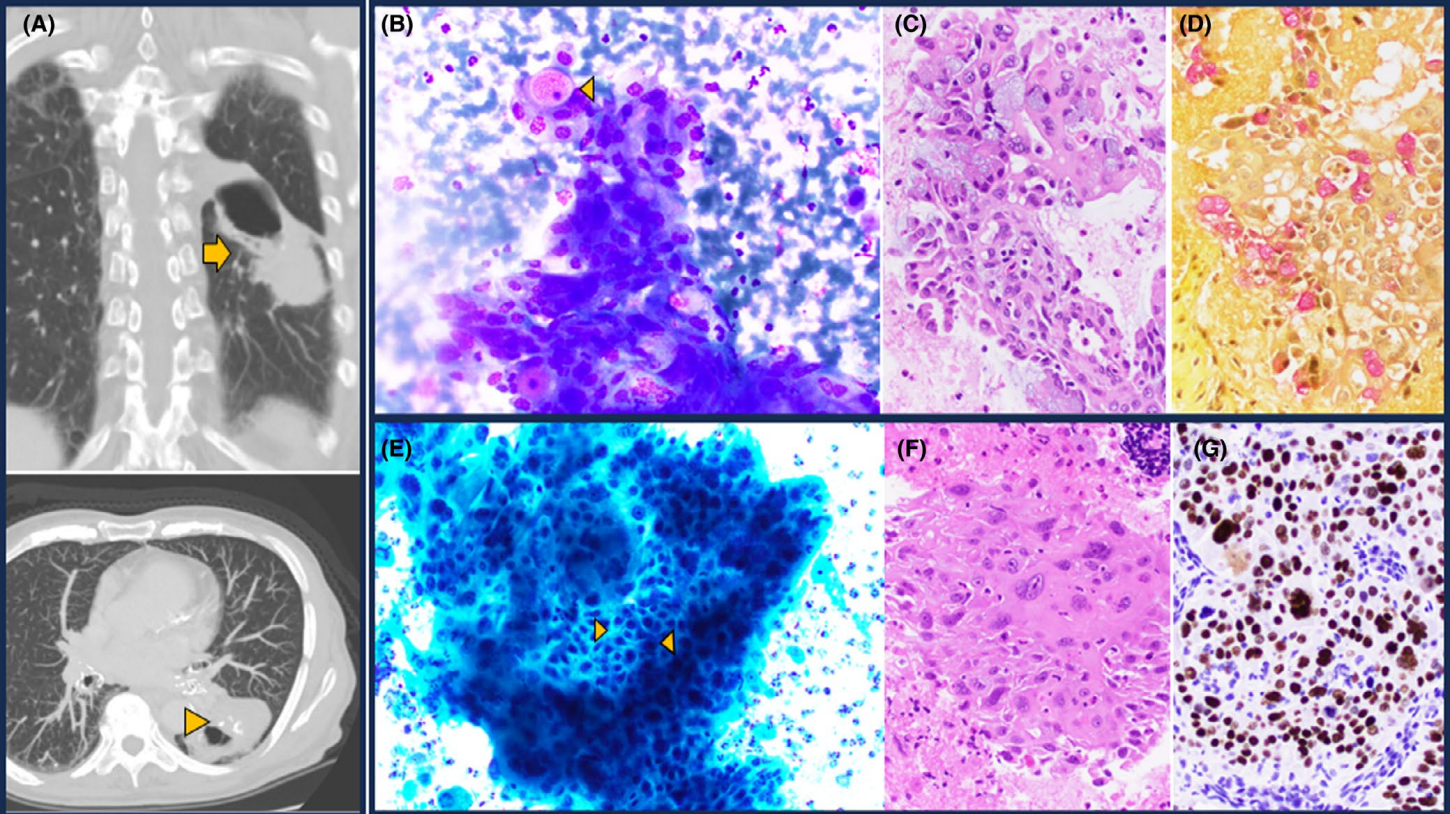
Adenosquamous carcinoma arising in bronchogenic cyst. (A) Imaging: CT scans reveal a large lesion with both cystic and solid parts (arrow), and calcifications at the transition point (arrowhead). (B–G) Fine‐needle aspiration. Upper Panel: Adenocarcinoma: The Diff‐Quick stain (B) displays cells with uniform nuclei and ample cytoplasm, including a cell with a large mucus vacuole. The cell block (C) depicts malignant glands with intracellular mucus, highlighted by the mucicarmine stain (D). Lower Panel: Squamous cell carcinoma: The Pap stain (E) shows prominent intercellular bridges (arrowheads). The cell block (F) contains keratinizing malignant cells that are positive for the P40 immunostain (G). (All images, 400×).

A never‐smoker 74‐year‐old female with rheumatoid arthritis was diagnosed with an IPBC 59 years earlier at age 15, following a bout of haemoptysis. She had been asymptomatic for the last 43 years. Surveillance was done with periodic imaging. She had no family history of lung cancer, no identifiable environmental toxic exposures and no history of previous radiotherapy. During routine rheumatology visits, unexplained dropping serum sodium levels ranging between 130 and 134 mmol/L (NR: 135‐145) were noticed. Diagnostic workup revealed enlargement of her IPBC and development of a solid component with calcifications, worrisome for malignancy (Figure 1A).

## 3 MATERIALS AND METHODS

A CT‐guided fine‐needle aspiration (FNA) of the mass was performed. Diff‐Quick and Papanicolaou stained smears and a cell block were prepared. A consent form allowing the use of surgically obtained material for research or educational purposes, provided that patient data remain anonymous, was signed by the patient.

## 4 RESULTS

**FIGURE 2:**
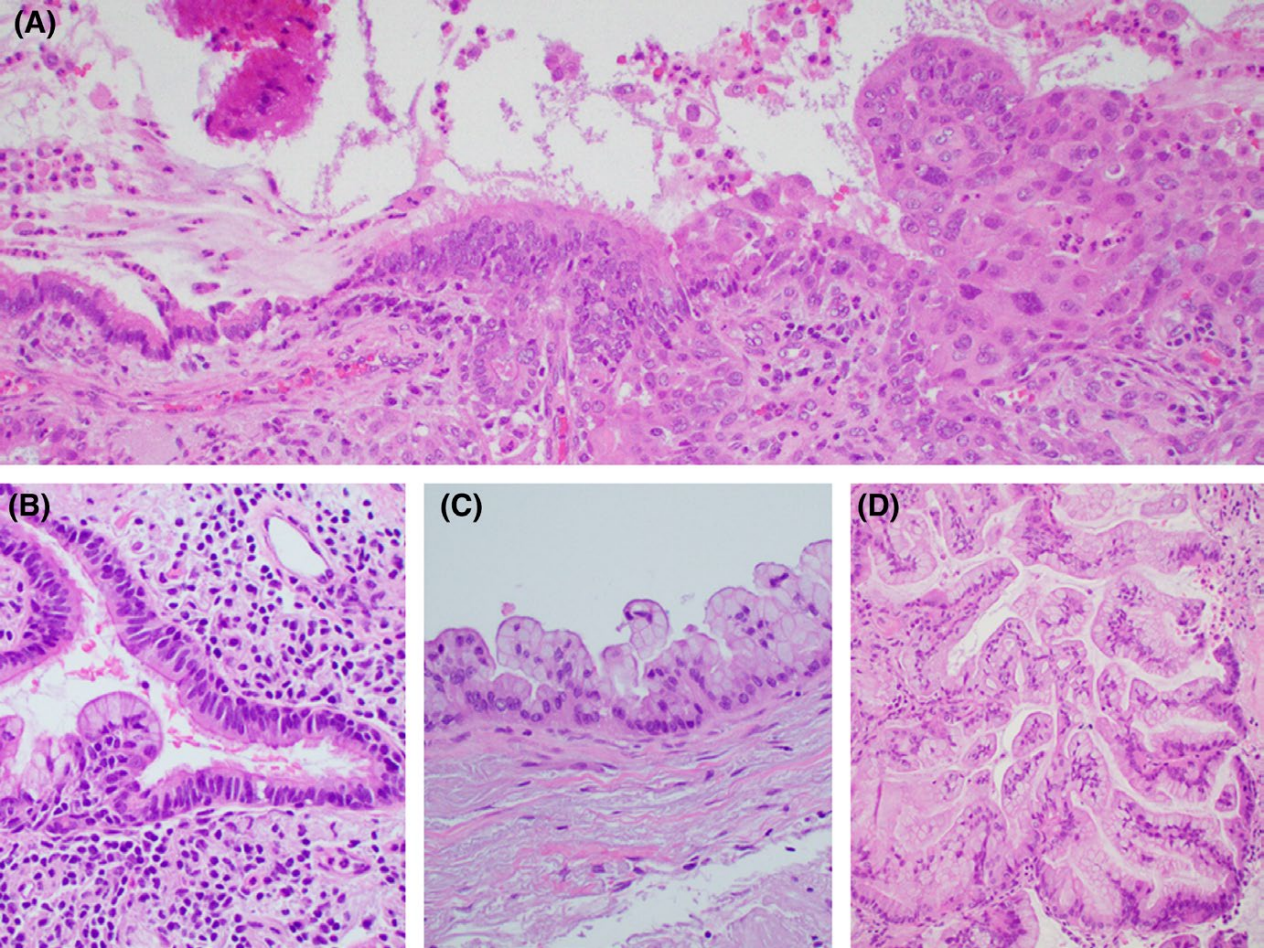
Adenosquamous carcinoma arising in bronchogenic cyst. (A) Pseudostratified ciliated respiratory epithelium of the cyst on the centre, transitions into a squamous cell carcinoma on the right and to dysplastic columnar glandular epithelium on the left (H&E, 20×). (B) Benign ciliated respiratory epithelium with focal mucinous metaplasia (H&E, 40×). (C) Cyst lining with borderline mucinous tumour showing bland cytology but abnormal papillary architecture (H&E, 40×). (D) Mucinous adenocarcinoma showing complex tubulopapillary architecture (H&E, 40×).

The samples showed tumour clusters with branching architecture, consisting of cells with abundant vacuolated cytoplasm and interspersed with cells containing large mucin vacuoles, indicative of glandular differentiation (Figure 1B,C). Additionally, there were groups displaying prominent intercellular bridges and dense cytoplasm, along with orangeophilia in the Papanicolaou stain, indicative of squamous differentiation (Figure 1E,F). The presence of both glandular and squamous components was corroborated by the mucicarmine stain and P40 immunostain (Figure 1D,G). The prognostic biomarker panel performed on the FNA was positive for a G12D *KRAS* mutation in exon 12, and negative for *EGFR, BRAF, ROS‐1* and *ALK* mutations/rearrangements. Staging PET/CT showed station 11L lymphadenopathy; however, endobronchial ultrasound FNA of mediastinal lymph nodes was negative. The patient underwent a left lower lobectomy with mediastinal lymph node dissection. A 5.5‐cm solid mass adjacent to a 4‐cm unilocular cyst was identified. Histologic examination showed an organ confined adenosquamous carcinoma intimately associated with an IPBC. Transition between the benign cyst lining and the malignant components was evident (Figure 2). Twenty‐two regional lymph nodes were negative. PD‐L1 22c3 tumour proportion score was 60% (high expression). Final AJCC stage was pT3N0. The patient received four cycles of adjuvant cisplatin/docetaxel and two cycles of atezolizumab. Immunotherapy was discontinued due to immune‐mediated colitis. She is in remission 2.4 years after surgery.

## 5 DISCUSSION

BCs are benign embryological malformations of the tracheobronchial occurring between the 5th and 16th week of gestation. They can detach and migrate to any site within the thoracic cavity and rarely outside.^1^ Intrapulmonary BCs (IPBCs) account for ~30% of all BCs and affect the lower lobes like in this patient.^4^ The prevalence of BCs has been estimated to be between 1 in 4200 to 6800 hospital admissions.^3^, ^4^

Histologically, the cyst walls recapitulate the normal components of the airways, being lined by respiratory epithelium with or without minor salivary glands, cartilage or smooth muscle.^1^, ^4^

The clinical presentation of BCs is diverse and related to size and location. Malignant transformation of a BC is very uncommon and estimated at <0.7%.^2^

The symptom signalling malignant transformation of this patient's long‐standing IPBC was persistent hyponatraemia. Hyponatraemia occurs in about 25% of all small and non‐small cell lung cancers, and it has been reported to correlate with increased mortality in prospective studies and meta‐analyses.^5^ Hyponatraemia is caused by the production of antidiuretic hormone‐like peptides by the tumour leading to an ectopic syndrome of inappropriate secretion of antidiuretic hormone, and severity correlates with the extent of disease. Hyponatraemia is typically detected during routine laboratory tests, but it may also cause neurological symptoms. Platinum‐based chemotherapy also induces hyponatraemia, which can increase the rate of complications when it coincides with cancer‐induced hyponatraemia.^5^

Adenosquamous carcinoma (ASC) comprises <3% of all non‐small cell carcinomas and is defined by the presence of at least 10% coexisting squamous cell carcinoma and adenocarcinoma components.^6^, ^7^ Keratinization and prominent intercellular bridges characterize the squamous component, and the presence of glandular lumens or mucinous secretions characterize the glandular component.^6^ Histochemical and immunohistochemical stains can be used to support morphology or to assign lineage when histology is unclear. Mucicarmine stain, thyroid transcription factor‐1 (TTF‐1) and Napsin A support an adenocarcinoma component and P40 supports squamous differentiation.^7^ When using ancillary tests, it is crucial to avoid overcalling benign entrapped lung, respiratory bronchioles and areas of bronchiolar/squamous metaplasia as malignant components. In the WHO Reporting System for Lung Cytopathology, ‘non‐small cell carcinoma NOS’ is the suggested term for limited biopsies showing distinct squamous and glandular components, with a note indicating the possibility of an adenosquamous carcinoma. Since a definitive classification requires that each component should constitute at least 10%, this is best determined in larger biopsies or resection specimens. ASC has been reported to have a more aggressive biological behaviour than single histology tumours of similar stage.^7^ A recent study using Laser Capture microdissection to conduct molecular profiling of several ASCs confirmed that both components have a clonal origin.^8^ A significant finding of that study was the high prevalence of potential therapeutic targets, which supports routinely performing molecular profiling for this subtype. Our case had a G12D *KRAS* mutation in exon 12, reported to be more common in adenocarcinomas from never‐smoker females and to have better progression‐free survival with conventional chemotherapy, but inferior responses to immunotherapy.^9^ Our patient was a never‐smoker female and she is in remission 2.4 years after treatment. Immunotherapy had to be discontinued due to the development of autoimmune colitis. She had rheumatoid arthritis and immune‐related adverse events have been reported to occur in up to 71% of patients with pre‐existent rheumatologic disease.^10^

Mucoepidermoid carcinoma (MEC) arising in BCs has also been described at various locations including the lung.^2^, ^3^ MECs also consist of glandular and squamous components; however, their diagnosis requires a tumour originating from peribronchial minor salivary glands, and/or a classic low‐grade component, and/or the presence of a *MAML2* rearrangement. By contrast, ASC is, by definition, a high‐grade tumour; does not originate from minor salivary glands; and does not have *MAML2* rearrangements.^6^, ^7^ Segregating MEC from ASC can be challenging in limited samples; potentially useful tests include TTF‐1 and Napsin‐A immunostains, which are typically negative in MECs but positive in some ASCs, *MAML2* rearrangements by FISH or NGS, since these are present in 67% of MECs but absent in ASCs, and *EGFR* mutations by PCR or NGS, which are detected in >30% of ASCs, but in <3% of MECs.

The risk of malignant transformation of BCs is estimated at less than 1%; however, the current recommendation for managing bronchogenic cysts is surgical excision^1^, ^2^ regardless of age or symptoms due to this risk. Malignant transformation can occur at any age, even 59 years after the initial diagnosis, as in this patient. For adult asymptomatic patients who opt for conservative management, close long‐term follow‐up is recommended.^2^

In summary, we present the first report of an intrapulmonary bronchogenic cyst that underwent malignant transformation to adenosquamous carcinoma. This case was identified during the workup of persistent hyponatraemia, a frequent but often overlooked symptom of lung cancer. Recent molecular analyses of ASCs indicate that they are not collision tumours, but rather represent divergent differentiation from a common precursor. Notably, ASCs often possess mutations that can be targeted, like lung adenocarcinomas, and in contrast to squamous cell carcinomas. Current guidelines recommend the excision of all BCs, irrespective of age or symptoms, due to the potential for malignant transformation, which as demonstrated in this case, may occur as late as 59 years after identification.

## AUTHOR CONTRIBUTIONS

AA was involved in original manuscript draft. SS and HC were involved in manuscript editing and critical revision. HM was involved in images and project administration.

## CONFLICT OF INTEREST STATEMENT

The authors do not have conflict of interest to declare.

## ETHICS STATEMENT

According to institutional guidelines, ethics committee approval is generally not required for case reports. Patients receiving care at the Indiana University School of Medicine sign a consent form allowing the use of surgically obtained material for research or educational purposes, provided that patient data remain anonymous.

## Data Availability

Data sharing is not applicable to this article as no new data were created or analyzed in this study.
